# Supplementary material for: Unsuccessful treatment outcome and associated risk factors. A prospective study of DR-TB patients from a high burden country, Pakistan
Source: PLoS One. 2023 Aug 10;18(8):e0287966. doi: 10.1371/journal.pone.0287966 (PMC10414635; doi:10.1371/journal.pone.0287966)
Supplement: Attachment — Submitted filename: Response to Reviewers.docx [file pone.0287966.s002.docx]

**Rebuttal Letter**

We are thankful to the esteemed editor/reviewer for his/her time and constructive comments, which has helped us in improving the quality of the manuscript. We have made changes according to editors’/reviewers’ comments and highlighted them in red text in the revised version of the manuscript. Here is our response to all the journal requirements and additional editor comments. We hope that our response to these comments will satisfy the editors/reviewer. If the reviewer /editorial team are not satisfied with the answers, we are open for further clarification.

**Journal Requirements:**

When submitting your revision, we need you to address these additional requirements.

1. Please ensure that your manuscript meets PLOS ONE's style requirements, including those for file naming.

**Response**: We have adhered to all the PlosOne styling requirements including file naming.

2. In your Data Availability statement, you have not specified where the minimal data set underlying the results described in your manuscript can be found. PLOS defines a study's minimal data set as the underlying data used to reach the conclusions drawn in the manuscript and any additional data required to replicate the reported study findings in their entirety. All PLOS journals require that the minimal data set be made fully available. Upon re-submitting your revised manuscript, please upload your study’s minimal underlying data set as either Supporting Information files or to a stable, public repository and include the relevant URLs, DOIs, or accession numbers within your revised cover letter.

**Response:** We have uploaded **supporting information file** along with revised manuscript submission and hope it will fulfill the minimum data availability requirement.

3. We note you have included a table to which you do not refer in the text of your manuscript. Please ensure that you refer to Table 4 in your text; if accepted, production will need this reference to link the reader to the Table.

**Response:** The said suggestion has been rectified and is highlighted in the revised manuscript version.

**Additional Editor Comments**

1. Lost-to-follow up (N=34) included in “unsuccessful treatment” group. Need to justify that or need to discuss the possible influence of that in results and conclusions

**Response:** In data collection section of methodology, loss to follow up patients have been grouped in “unsuccessful treatment outcome” which is inline with the WHO and national tuberculosis program (NTP), Pakistan guidelines. Similar grouping has been reported in the published literature as well [1, 2]. This is quite logical as loss to follow up DR-TB patients abort the treatment and hence ultimately impact the overall success rate (cured and completed) of the study and study site performance.

2. Abstract. “The mean age of patients was 36.75±15.69 years”. Suggest rewording because mean is a single number.

**Response:** Thanks for the suggestion and have been rectified in the abstract.

3. Authors say “All relevant data are within the manuscript and its Supporting Information files”. I haven’t seen person level data.

**Response:** Personal level data has been uploaded as **supporting information file.**

4. line 59-62. I cannot understand the incidence given as a percentage. What is the denominator? Is this the prevalence?

**Response:** Rightly pointed out. This statement needs to be revised as

**“**An estimated 4.2 % (95% confidence interval [CI] = 3.2–5.3%) of new TB cases and 7.3% (95%CI = 6.8–7.8) of previously treated TB cases had DR-TB in Pakistan as per WHO global TB report 2020”. And has been rectified in the revised manuscript.

5. I did not find the study aims within introduction section. It is needed to assess the appropriateness of methodology to achieve that aim. What this prospective study aimed to achieved? I had to rely on the brief version found in the abstract.

**Response:** Introduction section has been revised and additional information regarding the study rational has been added and we hope this will help to understand the aim of this prospective study.

6. Do you really need figure1? What additional info it provides than those in lines 160-162?

**Response:** As suggested by the esteemed reviewer, we have removed the figure as it appeared to be just information duplication already provided in the “Description of DR-TB patients” section.

7. Analyses presented are described multivariate, but they are actually multivariable.

**Response:** These are univariate logistic regression analysis and multivariate logistic regression analysis. These have been rectified in the revised manuscript version.

8. Line 111. “any comorbidity”. Did you use a specific definition for comorbidity here?

**Response:** No, there wasn’t any specific definition. Any prevailing disease along with DR-TB was recorded as comorbidity.

9. lines 155-157. Two sentences are contradictory to each other. This makes unclear how the variables in table 5 were identified. Comparison of tables 4 and 5 tells me only the 1st of these sentences is the correct one. It agrees with the cover letter too. So, I wonder about the 2nd sentence.

**Response:** P-value < 0.05 was used to describe statistical significance of any included variable in univariate and multivariate logistic regression analysis. However, it is evident in the published literature that in the presence of strong confounding, the significance-test strategies produced notably less bias when the alpha level was 0.20 rather than 0.05 and that significance-test strategies performed best when the p-value was set to a high value (i.e., 0.20 rather than 0.05)[3]. Additionally, already published articles in PlosOne [4] and the international journal of Tuberculosis and lung disease [5] has also included independent variables in univariate logistic regression analysis with p-value less than 0.2 to the multivariate logistic regression analysis. As these variables in univariate analysis have higher probability to be significant in multivariate logistic regression analysis. Furthermore, if reviewers still considers that variables with p-value < 0.05 in univariate logistic regression analysis should be eligible for multivariate logistic regression analysis, then we will re-analysis the data.

10. line 164. spellings. “bothe”

**Response:** Spelling rectified in revised manuscript.

11. Table1 footnote: remove everything that are not applicable to this table.

**Response:** Anything not applicable is removed from footnote.

12. Why “TB family history” is investigated as a possible factor in table1 when the TB is an infectious disease? By family history do you mean “those living with”, excluding ancestors?

**Response:** In table 1, information regarding “TB family history” was documented only as baseline socio-demographic and baseline clinical information and was meant to provide whether any close family member living in same vicinity had any infectious disease history.

13. line 189. “Cured and treatment completed” or “cured or treatment completed”?

**Response:** Successful treatment outcome include “cured and treatment completed”.

14. Table3. died=48. does this include deaths due to any reason (eg, road accident) or death due to TB only?

**Response:** Cause of death among DR-TB patients was either TB or clinical conditions due to TB disease progression i.e., cardiac arrest, Myocardial infarction or chronic illness.

15. Table3. defaulted=34. should this be ‘loss to follow up’?

**Response:** Yes, it should be Loss to follow Up and has been rectified in revised manuscript.

16. Table 4. this table not cited anywhere in the text. all the tables and figures must be cited even if they contain intermediate results.

**Response:** Noted and this table has been cited and rectified in revised manuscript.

17. Table 5. B and SE are redundant in the presence of OR, CI, and P-value.

**Response:** Noted. B and SE has been removed in revised table.

18. All available patients were included. But small sample size with only 84 unsuccessful outcomes compared to number of parameters in the model presented in table5 is a limitation.

**Response:** The sample size for assessing the management of DR-TB patients and the associated therapy outcomes within the current research was calculated on the basis of expected cases of DR-TB in new and retreated TB cases in 2016, which was 20% (95% CI 7.3-34)[6]. To calculate sample size, Daniel’s sample size calculation formula (Daniel and Wayne, 1995) was used as provided below:

n= Z^2^ P (1-P) / d^2^

where,

n= required sample size

Z= Z statistics to the level of confidence (For 95% level of confidence, Z=1. 96)

P= Predicted proportion (in proportion of one; for 20%, P=0. 20)

d= Precision (in proportion of one; if 5%, d= 0. 05)

A total of 246 patients was estimated as the sample size in the current study by simply substituting the values in the above formula. By incorporating the 10% dropout, the final sample size was calculated to be 272 DR-TB outpatient. So, in our opinion, sample size does not pose any limitation to the study. And similar outcomes have been reported in the literature.

**References**

1.	Javaid, A., et al., *Predictors of poor treatment outcomes in multidrug-resistant tuberculosis patients: a retrospective cohort study.* Clinical Microbiology and Infection, 2018. **24**(6): p. 612-617.

2.	Naz, F., et al., *High rate of successful treatment outcomes among childhood rifampicin/multidrug-resistant tuberculosis in Pakistan: a multicentre retrospective observational analysis.* BMC infectious diseases, 2021. **21**(1): p. 1-11.

3.	Maldonado, G. and S. Greenland, *Simulation study of confounder-selection strategies.* American journal of epidemiology, 1993. **138**(11): p. 923-936.

4.	Meshesha, M.D., *Predictors of sputum culture conversion time among MDR/RR TB patients on treatment in a low-income setting.* Plos one, 2022. **17**(11): p. e0277642.

5.	Ahmad, N., et al., *Management and treatment outcomes of MDR-TB: results from a setting with high rates of drug resistance.* The International Journal of Tuberculosis Lung Disease, 2015. **19**(9): p. 1109-1114.

6.	WHO, *Global tuberculosis report 2016*. 2016, World Health Organization: Geneva.
