# Supplementary material for: Unsuccessful treatment outcome and associated risk factors. A prospective study of DR-TB patients from a high burden country, Pakistan
Source: PLoS One. 2023 Aug 10;18(8):e0287966. doi: 10.1371/journal.pone.0287966 (PMC10414635; doi:10.1371/journal.pone.0287966)
Supplement: Attachment — Submitted filename: Response to Reviewers.docx [file pone.0287966.s003.docx]

**Rebuttal Letter**

Authors are thankful to the esteemed editor/reviewer for his/her time and constructive comments, which has helped us in improving the quality of the manuscript. We have made changes according to editors’/reviewers’ comments and highlighted them in red text in the revised version of the manuscript. Here is our response to all the journal requirements and additional editor comments. We hope that our response to these comments will satisfy the editors/reviewer. If the reviewer /editorial team are not satisfied with the answers, we are open for further clarification.

**Journal Requirements:**

When submitting your revision, we need you to address these additional requirements.

1. Please review your reference list to ensure that it is complete and correct. If you have cited papers that have been retracted, please include the rationale for doing so in the manuscript text or remove these references and replace them with relevant current references. Any changes to the reference list should be mentioned in the rebuttal letter that accompanies your revised manuscript. If you need to cite a retracted article, indicate the article’s retracted status in the References list and also include a citation and full reference for the retraction notice.

**Response**: We have adhered to all the PlosOne styling requirements including references and, in our knowledge, we haven’t included any retracted article reference. .

2. Authors have a good explanation for including lost-to-follow-up people in the unsuccessful group as explained in the rebuttal letter. This is a context specific explanation, and therefore the same explanation is invalid for most studies in other contexts. Therefore, authors’ explanation needs to be included in the manuscript for readers.

**Response:** Thanks for the suggestion and guidance. The said information has been added to the revised manuscript in data collection section of methodology.

3. I questioned authors’ description of mean age as “36.75±15.69 years”. Their response implies they haven’t understood the point I raised. For example, according to accepted mathematical standards, 10±3 is read as 10-3 or 10+3, meaning 7 or 13. Therefore, reporting mean age as 10±3, need to be read as mean age is 7 or 13. That does not make sense because mean age should be a single number. If you are reporting mean and SD (or mean and SE or mean and CI) what is presented should be explicit.

**Response:** Highly thankful for the extremely helpful and detail explanation regarding age. The sentence has been rectified in revised manuscript and it should be written as “The patient’s age (Mean ± SD was 36.75 ± 15.69 years” in abstract section. The said suggestion has been rectified and is highlighted in the revised manuscript version.

4. I questioned about presenting multivariable logistic regression results as multivariate logistic regression results. Authors’ response shows they haven’t understood the question. Question was about inappropriate terminology. I know multivariate and multivariable are interchangeably used in literature to mean the same think, but that is erroneous. I invite authors to know the difference between those two very different types of models, and not to present multivariable models as multivariate models.

**Response:** Authors are extremely thankful to esteemed reviewer**’s** suggestion for the use of correct terminology. Rightfully suggested, the said terminology “multivariate” has been rectified and more relevant and precise terminology “multivariable” has been used throughout in the revised manuscript.

5. Family history as a possible factor. Authors’ response was exactly what I assumed. This response needs to be in the manuscript to avoid the need for readers also to assume the same. I think the use of "family history" is a misleading term here.

**Response:** Thanks for the suggestion. Esteemed reviewer advised for the use of more relatable and correct use for “Family history”. Thus “family history” may be replaced with more appropriate term “**Close Family member with TB History**” as per our understanding. But after carefully going through the study, authors could not evaluate any significant importance to this information as rightly said by the reviewer in previous revision as “ Why “TB family history” is investigated as a possible factor in table1 when the TB is an infectious disease?”. Therefore, to avoid readers confusion and non-importance to the said information, we have removed the TB family History from the revised manuscript in Table 1. Still, authors are open if data regarding this term needs to be included or term to be modified.

6. Table 3. died=48. Authors’ response needs to be included in the manuscript for readers.

**Response:** Rightly pointed out. Cause of death has been explained in the revised manuscript. This statement needs to be revised as

**“**An estimated 4.2 % (95% confidence interval [CI] = 3.2–5.3%) of new TB cases and 7.3% (95%CI = 6.8–7.8) of previously treated TB cases had DR-TB in Pakistan as per WHO global TB report 2020”. And has been rectified in the revised manuscript.

7. I did not find the study aims within introduction section. It is needed to assess the appropriateness of methodology to achieve that aim. What this prospective study aimed to achieved? I had to rely on the brief version found in the abstract.

**Response:** Introduction section has been revised and additional information regarding the study rational has been added and we hope this will help to understand the aim of this prospective study.

8. Do you really need figure1? What additional info it provides than those in lines 160-162?

**Response:** As suggested by the esteemed reviewer, we have removed the figure as it appeared to be just information duplication already provided in the “Description of DR-TB patients” section.

9. Analyses presented are described multivariate, but they are actually multivariable.

**Response:** These are univariate logistic regression analysis and multivariate logistic regression analysis. These have been rectified in the revised manuscript version.

10. Line 111. “any comorbidity”. Did you use a specific definition for comorbidity here?

**Response:** No, there wasn’t any specific definition. Any prevailing disease along with DR-TB was recorded as comorbidity.

11. lines 155-157. Two sentences are contradictory to each other. This makes unclear how the variables in table 5 were identified. Comparison of tables 4 and 5 tells me only the 1st of these sentences is the correct one. It agrees with the cover letter too. So, I wonder about the 2nd sentence.

**Response:** P-value < 0.05 was used to describe statistical significance of any included variable in univariate and multivariate logistic regression analysis. However, it is evident in the published literature that in the presence of strong confounding, the significance-test strategies produced notably less bias when the alpha level was 0.20 rather than 0.05 and that significance-test strategies performed best when the p-value was set to a high value (i.e., 0.20 rather than 0.05)[1]. Additionally, already published articles in PlosOne [2] and the international journal of Tuberculosis and lung disease [3] has also included independent variables in univariate logistic regression analysis with p-value less than 0.2 to the multivariate logistic regression analysis. As these variables in univariate analysis have higher probability to be significant in multivariate logistic regression analysis. Furthermore, if reviewers still considers that variables with p-value < 0.05 in univariate logistic regression analysis should be eligible for multivariate logistic regression analysis, then we will re-analysis the data.

12. line 164. spellings. “bothe”

**Response:** Spelling rectified in revised manuscript.

13. Table1 footnote: remove everything that are not applicable to this table.

**Response:** Anything not applicable is removed from footnote.

14. Why “TB family history” is investigated as a possible factor in table1 when the TB is an infectious disease? By family history do you mean “those living with”, excluding ancestors?

**Response:** In table 1, information regarding “TB family history” was documented only as baseline socio-demographic and baseline clinical information and was meant to provide whether any close family member living in same vicinity had any infectious disease history.

15. line 189. “Cured and treatment completed” or “cured or treatment completed”?

**Response:** Successful treatment outcome include “cured and treatment completed”.

16. Table3. died=48. does this include deaths due to any reason (eg, road accident) or death due to TB only?

**Response:** Cause of death among DR-TB patients was either TB or clinical conditions due to TB disease progression i.e., cardiac arrest, Myocardial infarction or chronic illness.

17. Table3. defaulted=34. should this be ‘loss to follow up’?

**Response:** Yes, it should be Loss to follow Up and has been rectified in revised manuscript.

18. Table 4. this table not cited anywhere in the text. all the tables and figures must be cited even if they contain intermediate results.

**Response:** Noted and this table has been cited and rectified in revised manuscript.

19. Table 5. B and SE are redundant in the presence of OR, CI, and P-value.

**Response:** Noted. B and SE has been removed in revised table.

20. All available patients were included. But small sample size with only 84 unsuccessful outcomes compared to number of parameters in the model presented in table5 is a limitation.

**Response:** The sample size for assessing the management of DR-TB patients and the associated therapy outcomes within the current research was calculated on the basis of expected cases of DR-TB in new and retreated TB cases in 2016, which was 20% (95% CI 7.3-34)[4]. To calculate sample size, Daniel’s sample size calculation formula (Daniel and Wayne, 1995) was used as provided below:

n= Z^2^ P (1-P) / d^2^

where,

n= required sample size

Z= Z statistics to the level of confidence (For 95% level of confidence, Z=1. 96)

P= Predicted proportion (in proportion of one; for 20%, P=0. 20)

d= Precision (in proportion of one; if 5%, d= 0. 05)

A total of 246 patients was estimated as the sample size in the current study by simply substituting the values in the above formula. By incorporating the 10% dropout, the final sample size was calculated to be 272 DR-TB outpatient. So, in our opinion, sample size does not pose any limitation to the study. And similar outcomes have been reported in the literature.

**References**

1.	Maldonado, G. and S. Greenland, *Simulation study of confounder-selection strategies.* American journal of epidemiology, 1993. **138**(11): p. 923-936.

2.	Meshesha, M.D., *Predictors of sputum culture conversion time among MDR/RR TB patients on treatment in a low-income setting.* Plos one, 2022. **17**(11): p. e0277642.

3.	Ahmad, N., et al., *Management and treatment outcomes of MDR-TB: results from a setting with high rates of drug resistance.* The International Journal of Tuberculosis

Lung Disease, 2015. **19**(9): p. 1109-1114.

4.	WHO, *Global tuberculosis report 2016*. 2016, World Health Organization: Geneva.
