# Supplementary material for: Unsuccessful treatment outcome and associated risk factors. A prospective study of DR-TB patients from a high burden country, Pakistan
Source: PLoS One. 2023 Aug 10;18(8):e0287966. doi: 10.1371/journal.pone.0287966 (PMC10414635; doi:10.1371/journal.pone.0287966)
Supplement: Attachment — Submitted filename: Response to Reviewers.docx [file pone.0287966.s004.docx]

**Rebuttal Letter**

Authors are thankful to the esteemed editor/reviewer for his/her time and constructive comments, which has helped us in improving the quality of the manuscript. We have made changes according to editors’/reviewers’ comments and highlighted them in red text in the revised version of the manuscript. Here is our response to all the journal requirements and additional editor comments. We hope that our response to these comments will satisfy the editors/reviewer. If the reviewer /editorial team are not satisfied with the answers, we are open for further clarification. Additionally, we have learnt a lot from the esteemed reviewer for his thorough comments and suggestions for the quality of manuscript.

**Journal Requirements:**

When submitting your revision, we need you to address these additional requirements.

1. Please review your reference list to ensure that it is complete and correct. If you have cited papers that have been retracted, please include the rationale for doing so in the manuscript text or remove these references and replace them with relevant current references. Any changes to the reference list should be mentioned in the rebuttal letter that accompanies your revised manuscript. If you need to cite a retracted article, indicate the article’s retracted status in the References list and also include a citation and full reference for the retraction notice.

**Response**: We have adhered to all the PlosOne styling requirements including references and, in our knowledge, we haven’t included any retracted article reference. .

2. **Comment 1.** I had to spend some time to decide what it is first column. I realised in includes the number of people in the category with unsuccessful outcomes and that number as a percentage of the total number in the category (as opposed to total number in the category and of them % with unsuccessful outcomes). However, some of the presented univariable ORs are inconsistent with that. E.g., Residence variable. Authors need to check presented numbers.

**Response:** Thanks for the suggestion and guidance. The correction has been made in revised manuscript in the Residence category and all other variables are counter checked for correct numbers.

In Table 4, we had the option to present the unsuccessful treatment outcome in various ways in the first column . Considering e.g., **Gender variable** in both male and female categories we considered the number of unsuccessful treatment outcome and its percentage from the total number of males and females respectively. As, there were 132 females in study cohort. Out of 132 females, 91 had negative unsuccessful outcomes and 41 had positive unsuccessful outcome. So, we took the percentage of unsuccessful from total of 132 females in this category.

Its percentage could have been taken from the total number of unsuccessful outcomes of the study that is 84 or percentage of females with unsuccessful treatment comes out of the total study cohort population that is 271. **We are open to presenting the numbers, the way, the respected reviewer would suggest, if he is not satisfied.**

3. **Comment 2.** . Table4. Age variable. What is the meaning of 45/9% for >50 aged? Why age=50 people were not included? I noted they are included in table5.

**Response:** Thankful for pointing out the typo grammatical error. It is 45.9% and has been rectified in revised manuscript. Furthermore, age = 50 has been added and category is age ≥ 50 years as evident in table 5. It is rectified in revised manuscript.

4. **Comment 3.** Table4. No one was resistant to Eto. Did your SPSS software allow such a variable in logistic regression to derive P-value?

**Response:** Authors are extremely thankful to esteemed reviewer**’s** suggestion. Rightfully suggested, it seems illogical to include such a variable in statistical analysis to derive p-value, when none of the patient was resistant to Eto. The SPSS software presented non computable values and we put it as such. The said variable has been removed from table 4 in revised manuscript.

5. **Comment 4.** All tables, make sure number of decimal places are consistent.

**Response:** All the tables have been counter checked for inconsistencies for decimal and made corrections where needed.

6. **Comment 5.** Table5. How do you interpret the P-value reported for reference group in Baseline Sputum grading variable.

**Response:** This is incorrect. This should not be presented in multivariable table at all. We have removed it in a revised manuscript.
