# Supplementary material for: Unsuccessful treatment outcome and associated risk factors. A prospective study of DR-TB patients from a high burden country, Pakistan
Source: PLoS One. 2023 Aug 10;18(8):e0287966. doi: 10.1371/journal.pone.0287966 (PMC10414635; doi:10.1371/journal.pone.0287966)
Supplement: Attachment — Submitted filename: Response to Reviewers.docx [file pone.0287966.s005.docx]

**Rebuttal Letter**

Authors are thankful to the esteemed editor/reviewer for his/her time and constructive comments, which has helped us in improving the quality of the manuscript. We have made changes according to editors’/reviewers’ comments and highlighted them in red text in the revised version of the manuscript. Here is our response to all the journal requirements and additional editor comments. **We hope that our response to these comments will satisfy the editors/reviewer. If the reviewer /editorial team are not satisfied with the answers, we are open for further clarification**. Additionally, we have learnt a lot from the esteemed reviewer for his thorough comments and suggestions for the quality of manuscript.

**Journal Requirements:**

When submitting your revision, we need you to address these additional requirements.

1. Please review your reference list to ensure that it is complete and correct. If you have cited papers that have been retracted, please include the rationale for doing so in the manuscript text or remove these references and replace them with relevant current references. Any changes to the reference list should be mentioned in the rebuttal letter that accompanies your revised manuscript. If you need to cite a retracted article, indicate the article’s retracted status in the References list and also include a citation and full reference for the retraction notice.

**Response**: We have adhered to all the PlosOne styling requirements including references and, to our knowledge, we haven’t included any retracted article reference. .

2. **Comment 1.** In the last review I asked the meaning of P-value presented for the reference category in Baseline Sputum grading variable in table5. As the response to that, reference category has been removed from the table. The other 2 categories of that variable remain with their Odds ratios. That makes it unable to interpret those two odds ratios. Whenever category-specific odds ratios are presented for a multi-category variable, each odds ratio is interpreted relative to the reference category, therefore reference category should be explicitly mentioned, unless intuitive in the context. Can authors attend that please. Otherwise, the manuscript is very close to acceptance.

**Response:** Thanks for your suggestion and guidance throughout the manuscript review. And we learned a lot from esteemed reviewer comments during this process.

In the first place where the esteem reviewer asked, **“How do you interpret the P-value reported for reference group in Baseline Sputum grading variable”**. The incorporation of p-value over there was typographical error. But as the reviewer asked, due to confusion in understanding, we omitted the referent group from the table which was wrong thing to do. So, after going through to properly understand to find the error or query , we were able to explain the comment. In this category **negative sputum grading** was categorized as reference category as being the multi-category variable. Hope we are able to answer the raised point and in this process, we learned to review the manuscript with more care.
